# The Diagnostic Value of Hysterosalpingography and Hysterolaparoscopy for Evaluating Uterine Cavity and Tubal Patency in Infertile Patients

**DOI:** 10.7759/cureus.12526

**Published:** 2021-01-06

**Authors:** Soumya R Panda, B Kalpana

**Affiliations:** 1 Obstetrics and Gynaecology, All India Institute of Medical Sciences, Mangalagiri, IND; 2 Obstetrics and Gynaecology, Guru Hospital, Madurai, IND

**Keywords:** hysterosalpingography, hysterolaparoscopy, chromopertubation., hsg

## Abstract

Background and objective

Hysterosalpingography (HSG) is a common radiologic modality employed for the initial workup of female infertile patients to evaluate for tubal patency or any gross intrauterine pathology. HSG is a relatively cheap and easily available outpatient procedure but bears the risk of radiation exposure. The purpose of the study was to compare the diagnostic value of HSG with that of diagnostic hysterolaparoscopy in infertile women and to evaluate their role in the diagnosis and management of infertility.

Methodology

This study was carried out from February 2018 to January 2019. All women attending our outpatient department (OPD) for the treatment of infertility who were aged between 20-40 years were included in the study. Those with acute vaginal and cervical infection and those having an allergic reaction to the dye used in HSG were excluded from the study. Known cases of pelvic inflammatory disease and those who achieved pregnancy before the performance of hysteroscopy were also excluded from the study.

Results

A total of 172 women with primary or secondary infertility were enrolled in the study. Out of these, 13 women became pregnant, and two were lost to follow-up and were excluded from our study. Thus, a total of 157 infertile women were analysed. The agreement between HSG and hysteroscopy was 71.3% for the evaluation of the uterine cavity, and this was considered a fair strength of agreement between the procedures (k value=0.302).

Conclusion

Compared to HSG, the incidental findings detected by hysteroscopy amounted to 19.14%. Our study results depict the advantage of hysterolaparoscopy over HSG. Although the sensitivity of HSG is as good as that of diagnostic hysterolaparoscopy for the detection of tubal patency, a significant number of important incidental findings can be missed by it. Hence, diagnostic hysterolaparoscopy should be offered as the first-line modality for the evaluation of infertility wherever the procedure is available.

## Introduction

Infertility is a medical condition with significant psychological, social, and medical implications [1,2]. Tubal factor constitutes about 25-30% whereas uterine factors contribute to around 10-15% of infertile cases [3,4]. Hysterosalpingography (HSG) is a common radiologic modality employed for the initial workup of female infertile patients to evaluate for tubal patency or any gross intrauterine pathology. HSG is a relatively cheap outpatient procedure, but it bears the risk of radiation exposure. Though pelvic sonography and HSG are thought to be good modalities for evaluating uterine pathology, their ability in evaluating uterine cavity is limited to gross pathologies, and subtle changes in the form of small polyps, adhesions, and sub-endometrial myomas, which contribute to fertility, can be missed out by these procedures. Hysteroscopy, on the other hand, carries the advantage of having the ability to diagnose such subtle changes because of the magnification it possesses. Although hysterolaparoscopy is costlier and invasive, it is free from radiation exposure and provides a better real-time abdominopelvic view [5-7]. Additionally, hysterolaparoscopy not only detects the abdominopelvic pathologies but also can play a therapeutic role in the same setting [8,9]. Despite the above facts, the debate on the role of HSG versus hysteroscopy in the diagnosis and management of infertility still persists. The objective of this study was to evaluate the effectiveness of HSG in diagnosing tubal patency and uterine cavity abnormalities in comparison to diagnostic hysterolaparoscopy and to assess the role of hysterolaparoscopy in the comprehensive workup of infertility, which would help in implementing an appropriate management plan for the condition.

## Materials and methods

The present study was a prospective observational study conducted at the infertility clinic of the Department of Obstetrics and Gynecology, Guru Hospital, Madurai. The study was carried out from February 2018 to January 2019. All infertile women attending our outpatient department (OPD) who were aged between 20-40 years were included in the study. Those who were allergic to the dye used in HSG, those diagnosed with pelvic inflammatory disease or acute vaginal and cervical infection, or those who achieved pregnancy before undergoing hysteroscopy were excluded from our study.

An HSG was considered as normal if the following criteria were fulfilled: normality of uterine cavity; no evidence of tubal block; normal fallopian tube contour; and free bilateral spillage of contrast. HSGs or diagnostic hysterolaparoscopies were performed in all women under aseptic conditions after obtaining the informed written consent. If the HSG was found to be normal, hysteroscopy was performed only if the patient failed to conceive after three ovulation induction cycles. In patients with abnormal HSG findings, hysteroscopy was performed in the immediate postmenstrual phase. If the patient became pregnant within three ovulation induction cycles (before the performance of hysteroscopy), they were excluded from our study.

Sample size calculation and patient enrollment

Measuring the prevalence of infertility in India as 11%, with a two-sided significance level of 95%,and absolute precision of 5%, the sample size was calculated to be 152 [10]. The sample size was calculated using OpenEpi software version 3.01. The sample size was increased to 172 to allow for a potential dropout rate of around 15%. All HSGs and diagnostic hysterolaparoscopies were performed by specific but different individuals to decrease the chances of interobserver bias. A total of 172 women with primary or secondary infertility was enrolled in the study. Out of these, 13 women became pregnant, and two were lost to follow-up and were excluded from our study. Thus, a total of 157 infertile women were analysed.

Statistical analysis

Categorical variables were presented in numbers and percentages (%). The chi-squared test/Fisher's exact test was used for comparison of qualitative variables, Inter-rater kappa agreement was used to find the strength of agreement between the two methods. All statistical analyses were performed using the SPSS Statistics software version 21.0 (IBM, Armonk, NY).

## Results

A total of 157 subjects were analysed in this study. About 64.9% (102/157) of subjects had primary infertility and 35.1% (55/157) had secondary infertility. The average age and BMI of women in our study were 25.27 ±3.96 years and 25.84 ±5.44 Kg/m^2^, respectively. The mean duration of infertility was 2.90 ±1.74 years. These baseline characteristics are presented in Table 1.

**Table 1:** Baseline parameters SD: standard deviation; BMI: body mass index

Type of infertility	Primary infertility	Secondary infertility	P-value	Total cases
Number of patients	102	55	-	157
Age (years), mean ±SD	24.04 ±3.91	27.54 ±2.92	0.0001	25.27 ±3.96
BMI (Kg/m^2^), mean ±SD	25.43 ±5.78	26.61 ±4.64	0.1942	25.84 ±5.44
Duration of infertility (years), mean ±SD	2.88 ±1.66	2.94 ±1.87	0.8366	2.90 ±1.74

Regarding the uterine cavity findings, HSG was normal in 120 (76.43%) women and abnormal in 37 (23.57%) women; 105 (66.8%) women were found to have normal hysteroscopic findings and 52 women (33.2%) had abnormal hysteroscopic findings. A summary of the hysteroscopic and HSG findings is presented in Table 2.

**Table 2:** Evaluation of uterine cavity by HSG and hysteroscopy HSG: hysterosalpingography

Hysteroscopic findings	Number of patients (n=157)	Percentage (%)	HSG findings	Number of patients (n=157)	Percentage (%)
Normal cavity	105	66.8	Normal	120	76.4
Abnormal	52	33.2	Abnormal	37	23.6
Complete uterine septum	4	2.54	Filling defects	17	10.8
Partial septum	10	6.3	Irregular cavity	12	7.64
Asherman’s syndrome	7	4.45	Arcuate uterus	2	1.27
Bilateral ostia fibrosis	8	5.08	Bicornuate uterus	9	5.73
Unilateral fibrosis	4	2.54	Small cavity	4	2.54
Endometrial polyp	10	6.3			
Polypoidal endometrium	5	3.15			
Atrophic or pale endometrium	11	7			
Submucosal fibroid	4	2.54			
Endocervical polyp	2	1.27			
Cervical stenosis	12	7.64			

The comparison of uterine cavity findings on HSG and hysteroscopy is shown in Table 3. The sensitivity, specificity, positive predictive value, and negative predictive value were 42.3%, 85.7%, 59.45%, and 75% respectively. The agreement between HSG and hysteroscopy was 71.3%. This was statistically significant (p-value=0.002) with a fair strength of agreement between the two procedures [k value=0.302; standard error (SE) of kappa=0.080, 95% CI: from 0.145 to 0.460].

**Table 3:** Comparison of uterine cavity findings on HSG and hysteroscopy Sensitivity=42.3%; specificity=85.7%; positive predictive value=59.45%; negative predictive value=75%; kappa=0.302; standard error of kappa=0.080; 95% CI: from 0.145 to 0.460; strength of agreement is considered to be fair TP: true positive; TN: true negative; FP: false positive; FN: false negative; HSG: hysterosalpingography

		Uterine cavity findings on hysteroscopy		Total	P-value	Kappa
		Abnormal	Normal		0.0002	0.302
Uterine cavity findings on HSG	Abnormal	22 (TP)	15 (FP)	37		
	Normal	30 (FN)	90 (TN)	120		
Total		52	105			

A summary of the diagnostic hysterolaparoscopic findings is presented in Table 4. The tubal block was found in 38.2% of cases of primary infertility and 36.36% cases of secondary infertility.

**Table 4:** Diagnostic hysterolaparoscopy findings PID: pelvic inflammatory disease

Findings	Primary infertility (n=102)	Percentage (%)	Secondary infertility (n=55)	Percentage (%)
Tubal block	39	38.2	20	36.36
Tubal adhesions	10	9.8	12	21.81
Tubo-ovarian mass	2	1.9	8	14.54
Endometriosis	14	13.72	10	18.18
PID	11	10.78	15	27.27
Pelvic adhesions	10	9.8	14	25.45

The comparison of tubal status on HSG and diagnostic hysterolaparoscopy is shown in Table 5. The sensitivity, specificity, positive predictive value, and negative predictive value were 100%, 84.4%, 69.4%, and 100% respectively. The agreement between HSG and hysteroscopy was 88.5%. This was statistically significant (p-value=0.0001) with a good strength of agreement between the two procedures (k value=0.74; SE of kappa=0.056, 95% CI: from 0.631 to 0.841).

**Table 5:** Comparison of tubal patency detection on HSG and combined hysterolaparoscopy Sensitivity=100%; specificity=84.4%; positive predictive value=69.4%; negative predictive value=100%; Kappa=0.74; Standard error of kappa=0.056, 95% CI: from 0.631 to 0.841; the strength of agreement is considered to be good TP: true positive; TN: true negative; FP: false positive; FN: false negative; HSG: hysterosalpingography

		Tubal findings on hysteroscopy		Total	P-value	Kappa
		Abnormal	Normal		0.0001	0.74
Tubal findings on HSG	Abnormal	41 (TP)	18 (FP)	59		
	Normal	0 (FN)	98 (TN)	98		
Total		41	116	157		

## Discussion

In this study, we compared HSG with hysteroscopy considering uterine cavity and tubal factors separately. We found a fair strength of agreement for uterine cavity findings, whereas the strength of agreement between the two modalities was good for detecting the tubal blockage. HSG was normal in 76.43% of women and abnormal in 23.57%. Ibinaiye et al. [11] also found similar results (normal uterine cavity on HSG in 75.9% and abnormal in 24.1%). Chauhan et al. [12] and Vaid et al. [13] have reported a detection rate of 13% and 8.29% for abnormal uterine cavities on HSG. The most common abnormal uterine cavity finding on HSG in our study was filling defects (10.8%) followed by irregular uterine cavities (7.64%). Out of 157 women who underwent the hysteroscopic procedure, 66.8% were found to have normal hysteroscopic findings, with 33.2% of women having abnormal results. Wadhwa et al. [10] found abnormal findings in 35.51% whereas Chauhan et al. [12] reported abnormal findings in 20% of cases.

In the present study, the agreement between the two procedures was 71.3% for uterine cavity findings. In terms of comparison, the difference in findings between HSG and hysteroscopy was statistically significant (p-value=0.001) with a fair degree of agreement between the two procedures (k value=0.302). This means that a significant number of abnormal uterine cavity (around 29.7%) findings can be missed on HSG. Similar results were obtained in a recent study conducted by Wadhwa et al. [10], with 75% agreement between the two procedures.

There are varying data regarding the sensitivity and specificity of HSG in detecting uterine cavity abnormalities in different studies [8,14-15]. Thus, the sensitivity and specificity appear to range between 21-81 and 70-98%, respectively. Taşkın et al. [16] found a low sensitivity n their study, but this could be due to the fact that the male partners in most of the couples visiting their clinic had male factor infertility. The study by Nigam et al. [17] reported a positive predictive value of 70% with a false-negative rate of 12.96%.

We found incidental findings detected by hysteroscopy in 19.14% of cases. These incidental findings might have detrimental effects on fertility, and most of them such as partial septum, endometrial polyp, etc, could be easily treated by hysteroscopy. Other studies also found hysteroscopically detected incidental findings in 15.38% [10], 29.2% [16], 32.12% [13], and 13% [12] of cases.

Hysterolaparoscopy holds the advantage of being both a diagnostic and therapeutic procedure in various abdominopelvic pathologies including tubal pathologies. In the present study, we assessed the tubal patency in the female infertile patients by performing chromopertubation. We also compared the HSG with hysterolaparoscopy as the reference standard. We found a sensitivity of 100% and a specificity of 84.4% for the detection of tubal patency on HSG. Similarly, Agrawal et al. [18] found the sensitivity and specificity of HSG to be 100% and 52.31% respectively. On comparing the same parameters, Vaid et al. found a sensitivity of 80.6% and specificity of 81.5% for HSG. They found that the agreement between the two modalities was 74% [13]. Agrawal et al. [18] and Tognil et al. [19] found the probability of tubal occlusion on hysterolaparoscopy to be very low while HSG was normal. Similar results were found in the present study in that no patient showed tubal blockage on chromopertubation when HSG was reported to have tubal spillage of dye.

Study limitations and observations on future research

The only limitation of this study is that parameters indicating the success of fertility treatment such as clinical pregnancy rate and live birth rates were not studied in relation to hysterolaparoscopic findings. However, this is a separate domain and future research should be aimed at studying the correlation between hysterolaparoscopic findings and parameters such as clinical pregnancy rate and live birth rates, etc.

## Conclusions

Our study results illustrate the advantage of hysterolaparoscopy over HSG. Although the sensitivity of HSG is as good as that of diagnostic hysterolaparoscopy for the detection of tubal patency, a significant number of incidental findings can be missed by the procedure. Moreover, diagnostic hysterolaparoscopy holds the advantage of providing the details of other abdominopelvic pathologies, and it can also provide therapeutic intervention in the same setting. Hence, diagnostic hysterolaparoscopy should be offered as the first-line modality for the evaluation of infertility wherever the procedure is available. An abnormal HSG should always be confirmed with a hysterolaparoscopy.
